# Study design and protocol for moving forward: a weight loss intervention trial for African-American breast cancer survivors

**DOI:** 10.1186/s12885-015-2004-4

**Published:** 2015-12-29

**Authors:** Melinda R. Stolley, Lisa K. Sharp, Giamila Fantuzzi, Claudia Arroyo, Patricia Sheean, Linda Schiffer, Richard Campbell, Ben Gerber

**Affiliations:** 1 Cancer Center and Department of Medicine, Medical College of Wisconsin, 8701 Watertown Plank Road, Milwaukee, WI 53226-3548 USA; 2 Institute for Health Research and Policy, University of Illinois at Chicago (UIC), Chicago, IL USA; 3 Department of Pharmacy Systems, Outcome & Policy, UIC, College of Pharmacy, Chicago, IL USA; 4 Department of Kinesiology and Nutrition, UIC, College of Applied Health Sciences, Chicago, IL USA; 5 School of Nursing, Loyola University, Maywood, IL 60153 USA

**Keywords:** Breast cancer, Weight loss, African-American, Survivorship

## Abstract

**Background:**

Breast cancer survival rates are significantly lower among African-American women compared to white women. In addition, African-American women with breast cancer are more likely than white women to die from co-morbid conditions. Obesity is common among African-American women, and it contributes to breast cancer progression and the development and exacerbation of many weight-related conditions. Intervening upon obesity may decrease breast cancer and all-cause mortality among African-American breast cancer survivors.

**Methods/Design:**

Moving Forward is a weight loss intervention being evaluated in a randomized trial with a projected sample of 240 African American breast cancer survivors. Outcomes include body mass index, body composition, waist:hip ratio, and behavioral, psychosocial and physiological measures. Survivors are randomized to either a 6-month guided weight loss intervention that involves twice weekly classes and text messaging or a self-guided weight loss intervention based on the same materials offered in the guided program. The guided intervention is being conducted in partnership with the Chicago Park District at park facilities in predominantly African-American neighborhoods in Chicago. Recruitment strategies include direct contact to women identified in hospital cancer registries, as well as community-based efforts. Data collection occurs at baseline, post-intervention (6 months) and at a 12-month follow-up.

**Discussion:**

This study evaluates a community-based, guided lifestyle intervention designed to improve the health of African-American breast cancer survivors. Few studies have addressed behavioral interventions in this high-risk population. If successful, the intervention may help reduce the risk for breast cancer recurrence, secondary cancers, and co-morbid conditions, as well as improve quality of life.

**Trial registration:**

U.S. Clinicaltrials.gov number: NCT02482506, April 2015

## Background

Breast cancer is the second leading cause of cancer death among African-American women [1]. Despite lower incidence, breast cancer mortality rates for black women are higher than those for women of other racial/ethnic groups even after controlling for age, socioeconomic status, tumor stage and histology, hormone receptor status, and menopausal status ([2, 3], http://www.seer.cancer.gov/). Ninety- two percent of white women will survive for at least five years after diagnosis, compared to only 77 % of black women [4]. Additionally, African-American women with breast cancer are more likely to die from co-morbid conditions, including diabetes and hypertension [5, 6]. These disparities are not easily explained and involve multiple issues related to social determinants of health [7]. However, obesity and behavioral factors are likely additional and related contributors.

Obesity contributes to breast cancer progression, as well as the development and exacerbation of many co-morbid conditions [8–13]. This relationship remains after adjusting for stage at diagnosis, nodal status, treatment type, and menopausal status prior to diagnosis [8, 14–16]. Obesity is hypothesized to promote tumor progression by (1) producing higher concentrations of estrogen and testosterone [2, 17, 18], (2) contributing to insulin resistance, leading to increased levels of insulin-like growth factor-I (IGF-1) and insulin-like growth factor-binding protein-3 (IGFBP-3) [10, 19, 20], and (3) contributing to chronic inflammation [21]. This paper describes the study protocol for Moving Forward: A weight loss program for African-American breast cancer survivors.

Eighty-two percent of African-American women are overweight or obese [22] and many have dietary and physical activity patterns that contribute to obesity, cancer and other health risks [11, 23–26]. Among breast cancer survivors, baseline dietary data from the Women’s Healthy Eating and Living Study showed that African-American survivors (*N* = 118) consumed significantly more calories from fat and less fruit than Asians or whites, and they were less likely than other women to meet guidelines for physical activity [27]. Findings from a survey of 468 African-American breast cancer survivors documented that most did not exercise regularly, and median television viewing was over 5 h daily [28]. The combined effects of obesity, unhealthy diet and inactivity may contribute to the disparity in breast cancer survival among African-American women and may be the easiest modifiable factors to address in the near term [12, 29].

Several weight loss interventions report beneficial results for breast cancer survivors, including weight loss [30–34], prevention of weight gain [35], improved body composition and lipids [33, 36], decreases in sex hormones [36], decreases in dietary fat intake [35], increases in fruit, vegetable and/or fiber intake [35], increased physical activity [30, 35] and improved psychological status [35]. Inclusion of African-American women in these trials was limited. Considering the high rates of breast cancer and all-cause mortality, co-morbidities, and obesity, weight loss is an important goal. However, due to a complex interaction of behavioral, cultural and societal factors, African-American women are less likely to participate in traditional weight loss programs, more apt to drop out, and lose less weight than white women [37, 38].

Recent efforts support the feasibility of weight loss interventions for African-American breast cancer survivors [31, 39, 40] However, studies were not fully powered and none examined the physiological impact of weight loss for African-American breast cancer survivors. Weight loss trials with white breast cancer survivors support the positive impact of weight loss on intermediate markers of breast cancer including sex hormones (estrogen, estradiol, testosterone, sex hormone binding globulin), chronic inflammation (C-reactive protein [CRP], interleukin-6 [IL-6], and TNF-α), and hyperinsulinemia (nsulin-like growth factor-1 [IGF-1], insulin-like growth binding protein-3 [IGBP3]). These data, along with those for body composition (percent body fat vs lean mass), are particularly important for African-American survivors given the historically low levels of weight loss observed in interventions. Gathering body composition and biological data will enhance our understanding of how weight loss, even if minor amounts, may impact breast cancer recurrence risk and overall health risk among African-American women.

## Methods/Design

### Study design

Moving Forward is a randomized trial that examines the effects of a culturally tailored guided weight loss program as compared to a self-guided weight loss program on the BMI, body composition and waist:hip circumference of 240 overweight/obese African-American breast cancer survivors. Diet and physical activity patterns, intermediate markers of breast cancer recurrence (i.e., sex hormones, markers of chronic inflammation and hyperinsulinemia), fatigue and quality of life will also be examined.

**Fig. 1:**
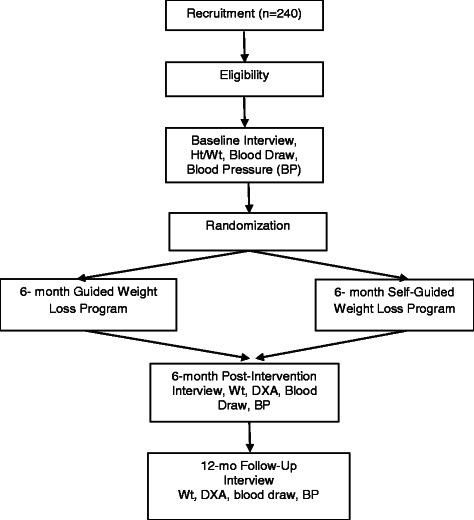
Study Design

**Fig. 2:**
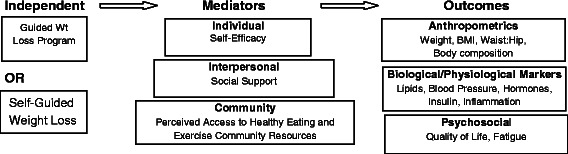
Study Conceptual Framework. The independent variable is group assignment. It is expected that outcome results will be mediated by self efficacy at the individual level, social support at the interpersonal level and perceived access to healthy eating and exercise resources at the community level

The study is based in eight Chicago Park District facilities located within communities that are predominantly African-American. Figure 1 provides an overview of the study design. Figure 2 provides an overview of the conceptual framework for the study.

### Ethics

The study procedures were reviewed and approved for ethical treatment of human subjects by the University of Illinois at Chicago Institutional Review/Ethics Board.

### Procedures

Women who respond to recruitment efforts complete a brief telephone interview to verify eligibility. Next, confirmation of stage I, II, or III breast cancer and approval for participation in a weight loss program that includes moderate physical activity is sought from the participant’s primary care physician (PCP) and/or oncologist. Once eligibility is established, participants are asked to come in for an interview and physical assessment. At the beginning of this appointment, staff meets individually with each participant to obtain written informed consent. They then complete a 75-min in person interview, a blood draw, blood pressure measurement, dual energy x-ray (DXA), and height and weight measurements. All participants are also asked to wear an accelerometer for 7 days during waking hours. Participants receive a $20 incentive for the interview, $10 for the blood draw, $10 for the DXA and $10 for wearing the accelerometer. Subsequently, participants are randomly assigned to one of two 6-month interventions: (1) Moving Forward guided weight loss intervention (MFG) or (2) Moving Forward self-guided weight loss intervention (SG). Participants once again complete the entire assessment process post-intervention and at a 12-month follow-up.

### Recruitment

Recruitment efforts include a number of different strategies. The most effective strategy relies on patient lists from cancer registries of three academic hospitals. Patients who were diagnosed with stages I, II, or III at least 6 months prior receive letters describing the study, followed by phone calls to assess interest and screen for eligibility. In addition, community-based efforts revolve around a number of community and institutional partners, including breast cancer support organizations, local churches, block clubs, community centers and community leaders. We also post notices within on-line communities that serve the African-American community.

### Eligibility

Inclusion criteria: (1) self-identify as Black or African-American (including individuals who are bi-racial but identify themselves as Black or African-American); (2) female; (3) Stage I, II, or III invasive breast carcinoma; (4) completed treatment (surgery, chemotherapy and/or radiation) at least six months *prior to* recruitment (ongoing treatment with adjuvant endocrine therapies is acceptable); (5) age 18 or above at time of diagnosis; (6) Body Mass Index (BMI) at least 25 kg/m^2^ – chosen because this includes only those participants who are overweight and would not be harmed by a 7 % weight loss; (7) physically able to participate in a moderate physical activity program as assessed by self-report and PCP/oncologist approval; (8) agree to random assignment and data collection; and (9) able to attend twice weekly classes for six months.

Exclusion criteria: (1) plans to move from the community during the study; (2) currently pregnant, less than 3 months post-partum, or pregnancy anticipated during the study; (3) taking weight loss medication prescribed by a doctor; (4) participation in another structured weight loss program that uses special foods; or (5) weight loss surgery in the past year, or planned weight loss surgery in the next year.

### Measures

#### Demographics

Demographic data include name, address, date of birth, marital status, number of children, education, occupational status, annual income, and insurance status.

#### Breast cancer treatment history

Diagnosis and treatment history (e.g., chemotherapy, radiation, previous and current adjuvant endocrine therapies) are collected from the treating oncologist and will be used for descriptive purposes only.

#### Co-morbid conditions

Respondents review a list of sixteen health problems (i.e., hypertension/high blood pressure, diabetes, arthritis) and report if they have ever been told by a doctor that they have this particular condition.

### Mediating variables

#### Social support for eating and exercise

This questionnaire asks respondents to rate on a five point scale (1 = never, 5 = very often) how often friends and family have done or said certain things related to the respondents’ efforts to change their dietary or exercise habits. Social support for eating habits is measured using two five-item subscales (encouragement and discouragement), each calculated separately for friends and family. Internal consistency coefficients range from 0.73 to 0.87. Social support for exercise is measured using one 10-item scale, also calculated separately for family and friends [41].

#### Self-efficacy for eating and exercise behaviors

The Physical Activity and Nutrition Self-Efficacy scale is an 11-item instrument that assesses the participant’s level of confidence that she can complete particular activities that promote weight loss [42]. This scale has adequate reliability, internal consistency, and construct validity, as well as good predictive validity among African-Americans.

#### Perceived access to healthy eating and exercise

These items come from the Robert Woods Johnson Active Where study [43]. Respondents rate their level of agreement (from 1 = strongly agree to 4 = strongly disagree) to four statements related to access to physical activity resources, five statements related to healthy eating resources, and five statements about perceived neighborhood safety. All scales show good internal consistency, with Cronbach’s alphas from 0.78 to 0.94 [43].

### Outcomes

#### Weight outcomes

##### Body Mass Index (BMI)

Height (baseline only) is measured to the nearest 0.1 cm using a portable stadiometer (Seca). Weight is measured to the nearest 0.1 kg using a digital scale (Tanita), with participants wearing light clothes without shoes. Two measurements for height and weight are taken. If there is a discrepancy of more than 0.5 cm for height or 0.2 kg for weight between the first and second measurements, a third measurement is taken. The mean of the two most closely aligned measurements is used to calculate BMI (weight (kg)/height (m)^2^.

##### Waist to hip ratio

Is measured with participants standing without outer garments and with empty pockets. Waist is measured to the nearest 0.1 cm at the level midway between the lower rib margin and the iliac crest, with the participant breathing out gently. Hip is recorded as the maximum circumference over the buttocks. Two measurements are taken. If there is a discrepancy of more than 1 cm, a third measurement is taken. The mean of the two measurements most closely aligned is used for analyses.

##### Body composition: Dual Energy X-ray Absorptiometry (DXA)

DXA allows quantification of the amount of adipose tissue located within the abdominal area and also throughout the entire body of each participant. DXA provides precise, non-invasive measures of fat mass and lean tissue mass (total body, as well as regional) [44]. This method is rapid, requires minimal effort from study participants and compares favorably with hydrostatic weighing for measurement of body fat percentages [45]. DXA measurements of participants’ total and regional fat and lean mass is conducted using the DXA Hologic 4500 W elite.

#### Behavioral outcomes

##### Diet

Our goal is to determine group means for consumption of energy, fruits and vegetables, fat, and fiber. A semi-quantitative Food Frequency Questionnaire (FFQ) is the most appropriate tool in this case [46–48]. The Block 2005 FFQ [49] estimates the usual intake of a wide array of nutrients and food groups, and allows for calculation of the Healthy Eating Index (HEI). Reliability and validity are established for the measure in a wide range of age, gender, income, and ethnic groups [50, 51].

#### Physical activity (self-report and objective)

##### Modified activity questionnaire

Kriska and Caspersen [52] The Modified Activity Questionnaire assesses self-reported leisure activity and television viewing. For leisure activity, respondents review a list of 17 popular activities (e.g., walking, gardening) and select those that they performed on at least 10 occasions in the last year. Participants are also given an opportunity to report leisure activities that are not on the list. Respondents then provide information on average frequency and duration for each activity. Responses are used to calculate the number of hours/week the participant engages in moderate and vigorous activity, along with total MET-hours per week. The questionnaire also asks how many hours per day the participant usually spends watching television. This activity questionnaire has been used in many large studies with diverse samples, including cancer survivors [53], and has well-established reliability and validity [52].

##### Godin leisure-time exercise questionnaire

This brief questionnaire asks how many times in a typical 7-day period the participant performs strenuous, moderate, or mild exercise for more than 15 min during her free time. These responses are used to calculate a leisure activity score and to classify the respondent as active, moderately active, or insufficiently active [54].

##### Accelerometer

The limitations of self-reported physical activity are well established [55]. Therefore, the ActiGraph GT3X activity monitor is used to obtain an objective measure of physical activity. The ActiGraph is a small, lightweight accelerometer designed to detect normal body motion. Participants are asked to wear the ActiGraph during waking hours for seven days. Only days on which the participant wore the accelerometers at least 10 h are included, and participants with fewer than four valid days are excluded from analyses. Thresholds suggested by Troiano and colleagues will be used to calculate the amount of time spent in moderate and vigorous physical activity [56, 57].

#### Biological/physiological outcomes

##### Biomarkers of breast cancer recurrence risk

A fasting blood sample is drawn according to standard procedures by a clinical research center phlebotomist. We chose markers for three proposed mechanisms by which obesity may contribute to breast cancer progression: altering levels of sex hormones (markers: estradiol, estrogen, sex hormone-binding globulin, testosterone), hyperinsulinemia (markers: IGF-I, IGBP3, C-peptide), and chronic inflammation (marker: C-reactive protein, IL-6) [58]. Staff at the clinical research center processes the blood samples according to standard procedures for storage. Briefly, a total of 30 ml of blood is collected in red top vacutainer tubes (without anticoagulant). Blood (approximately 15 ml) is allowed to clot for 20 min and subsequently centrifuged at 2500 rpm for 20 min to separate serum. Staff collects, aliquots serum, which is frozen at-80 °C until analysis. All breast cancer biomarkers are measured using commercially available ELISA kits (R&D Systems and Alpco). Each sample is assayed in duplicate and repeated if variability exceeds 15 %. We will monitor quality control of laboratory tests by ensuring internal positive and negative controls are within the parameters of the test kit for each assay and by evaluating for trends over time.

##### Biomarkers of Co-morbidities

Some of the collected blood is used to examine lipid profiles (HDL, LDL, triglycerides) as a marker of dyslipidemia and Hemoglobin A1c as a marker of impaired glucose tolerance. Individuals with an A1c level at or above 6.5 % who do not report a history of diabetes are informed of the result, provided counseling, and encouraged to follow up with their primary care providers. Hypertension is assessed by measuring diastolic and systolic blood pressure with an OMRON IntelliSense blood pressure monitor using a standard protocol.

#### Psychosocial outcomes

##### Quality of life

The patient-reported outcomes measurement information system (PROMIS) Global Health measure [59] consists of 10 self-reported global health items selected as an efficient way to gather general perceptions of health. The PROMIS items assess 5 domains: physical function, pain, fatigue, emotional distress, and social health. Two dimensions representing physical and mental health underlie the global health items in PROMIS. These global health scales can be used to efficiently summarize physical and mental health in patient–oriented studies.

##### Fatigue

The Brief Fatigue Inventory is a reliable nine-item instrument that uses 0–10 numeric rating scales to evaluate severity of fatigue. A global fatigue score is calculated by taking the mean of the 9 items [60].

##### Symptom checklist

The Breast Cancer Symptom Checklist (BCSCL) was originally developed for the Breast Cancer Prevention Trial and has been validated with a variety of breast cancer populations, including survivors [61]. Respondents are asked if they have experienced any of 17 listed symptoms and then rate the severity on a 5-point Likert-type scale from 0 (not at all bothered) to 4 (extremely bothered). This measure provides scale scores for eight clusters of symptoms: cognitive/mood symptoms, musculoskeletal pain, vasomotor symptoms, nausea, sexual problems, bladder problems, arm problems and body image. We modified the measure and did not include the nausea and sexual problems scales based on advisory board feedback.

### Intervention

Participants are randomized to either the Moving Forward Guided program (MFG) or the Moving Forward Self-Guided program (SG) for six months. MFG includes in-person classes and text messaging, while the SG includes a curriculum manual with handouts related to the intervention topics, but no classes or text messaging.

#### Moving forward

The intervention was developed based on formative qualitative work with African-American breast cancer survivors, followed by a pilot of the guided program that led to further refinements [30, 62].

#### Theoretical framework

The Moving Forward intervention integrates concepts from Social Cognitive Theory (SCT) [63] and the Socio-Ecological Model (SEM) [64, 65] to promote behavior change. SCT suggests that behavior can be explained by the dynamic interaction between behavior, personal factors (e.g., self-efficacy), and the environment (e.g., social support [66–70]. The intervention also incorporates tenets of the socio-ecological model (SEM) [64, 65], a model that goes beyond individual-level variables and emphasizes that support from the larger social context is needed for long-term behavior change [71]. Accordingly, SEM posits that weight status, diet, and physical activity are influenced by individual (e.g., beliefs, taste preferences), interpersonal (e.g., social support, traditions and role expectations), and community factors (e.g., access to resources that support health promotion) [72]. Interventions hoping to promote long-term behavior change must address these three levels of influence [73, 74]. Moving Forward accomplishes this by addressing: (1) Individual factors - acknowledging heavier body image ideals, identifying and addressing personal barriers to behavior change; (2) Interpersonal - the importance of food in the African-American culture and finding ways to integrate this value with healthful eating; providing low-fat versions of culturally traditional “soul food” recipes; acknowledging and addressing family roles and family resistance/support to change; providing information on integrating healthful lifestyle practices for the family; facilitating social support for making changes in diet, physical activity, and weight; understanding the important role of religion and worship in the women’s lives and how it affects their health perspectives and (3) Community - incorporating a sustainable link to a community physical activity resource that can address barriers to regular physical activity (i.e., safety, weather, access); problem solving around cost and availability of healthy food; introducing participants to unfamiliar community resources. Interestingly, a positive sense of community (e.g., social bonds between individuals and between individuals and their community) is associated with self-efficacy for physical activity among African-American women [75].

#### Intervention goals

The overall goal of Moving Forward is to make changes in health behaviors to promote a healthy weight. The weight loss goal is 7 % of baseline body weight (1–2 lbs. per week), consistent with the recommendations of an expert panel at National Institutes of Health [76]. Dietary goals aimed at producing weight loss, decreasing breast cancer recurrence risk, and improving overall health include: (1) a decrease in daily caloric intake (based on weight in pounds X 12 caloriesl/day, with 500–750 cal subtracted to create an energy deficit); (2) a decrease in dietary fat consumption to 20 % of total calories; (3) an increase in fruit and vegetable consumption to 7 daily servings; and (4) an increase in fiber to 25 g per day. For exercise, participants are advised to gradually increase their activity to a minimum of 180 min per week at 55–65 % maximal heart rate.

#### Moving Forward Guided Program Structure (MFG)

The MFG program meets twice a week for 26 weeks (see Table 1 for weekly themes) and is led by a team that includes a community dietitian, a community cancer exercise instructor, and a health psychologist. The program is conducted in city park district facilities, where participants enjoy free memberships, ongoing access to classes and fitness rooms, and the opportunity to maintain contact with program participants once the program concludes. The first meeting each week includes a 60-min class that addresses knowledge (e.g., relationship between obesity and breast cancer; food label reading; portions; available healthy living community resources), attitudes (e.g., pros and cons of weight loss; understanding the roles that food plays in one’s life; the concept of “fail to plan, plan to fail”) and cognitive behavioral strategies including self-monitoring of weight, food and physical activity; realistic goal setting; stimulus control; problem solving; mindfulness; cognitive restructuring and relapse prevention. These classes are led by a dietitian, a psychologist and a certified cancer exercise trainer. Table 1 provides a list of weekly curriculum topics. Pilot data showed that many women entered the program with low levels of knowledge about healthy eating and exercise. Thus, the first weeks are devoted to teaching core concepts (e.g., concept of calories in/out, food label reading, measuring heart rate), while later weeks are focused on cognitive-behavioral aspects of weight loss such as stimulus control, habit and mindfulness. Class activities include weighing in weekly; completing a food and activity self-monitoring record; increasing awareness of portions by weighing and measuring foods according to one’s typical portions and then according to recommended portions; creating stimulus control plans for home, car and work; identifying barriers to healthy eating and/or exercise and problem solving within small groups; going on a field trip to a local grocery store to practice reading food labels; creating an eating-out management plan; and identifying high-risk situations and brainstorming ways to manage them. The first weekly meeting also includes a support “icebreaker” (share the funniest moment of your breast cancer journey; what has been the most frustrating; etc.) and a 60-min exercise class taught by a certified cancer exercise trainer.

**Table 1:** Moving forward weight loss program weekly topics

Week 1	Introduction to program
Week 2	Self-monitoring and goal setting
Week 3	Using self-monitoring tools to make better choices
Week 4	Energy requirements
Week 5	Reading food labels and monitoring heart rate
Week 6	Measuring portions
Week 7	Breakfast and water – 2 key tools to losing weight
Week 8	Healthy grocery shopping
Week 9	Meal planning
Week 10	Holiday eating (scheduled according to when holiday falls)
Week 11	Stimulus control
Week 12	Mindful eating
Week 13	Eating away from home – restaurant and party strategies
Week 14	Program review – where were you, where are you now?
Week 15	Building movement into your daily life
Week 16	Barriers to healthy eating and exercise
Week 17	Problem solving
Week 18	The power of habit
Week 19	Strategies to increase fruits and vegetables
Week 20	Where you were, where you are and where you plan to go
Week 21	Relapse preventioin I – what is a lapse vs relapse
Week 22	Relapse prevention II – identifying high risk situations
Week 23	Relapse prevention III – maintaining a physically active lifestyle
Week 24	Relapse prevention IV – motivation to maintain changes
Week 25	Transitioning from Moving Forward to being on your own
Week 26	Graduation

The second weekly meeting is a 60-min exercise class taught by a cancer exercise trainer. The exercise classes incorporate a variety of activities, including traditional aerobics, line dancing, African dance, salsa, yoga, Pilates, and strength and flexibility training. Class time is also spent learning to use the park district fitness facility equipment to ensure that women feel comfortable and competent on the equipment, thus promoting enhanced self-efficacy and mastery of new skills. Many participants enter the program at very low levels of fitness; therefore, physical activity levels are increased gradually with special attention to concerns such as lymphedema and balance. Increased physical activity outside of class is encouraged by suggesting enrollment in additional local exercise classes, providing safe outdoor walking routes, and alerting women to activity resources online and on FitTV.

Participants often need further support and reinforcement of lifestyle changes outside of class, as well as timely information related to healthy eating and exercise resources. To provide this efficiently and effectively, MFG uses text messaging, a strategy successfully used in previous weight loss interventions with low income African-American women [77, 78]. A custom software application, mytapp, allows the psychologist and trainers to schedule two text messages each week to be sent to participants. Messages are less than 200 characters in length and are written to be brief, clear and motivational. The intent is to reinforce concepts covered in class while also supporting self-efficacy, social support and perceived access. For example, during the week that the intervention covers portions, participants receive a text that reads, “Are you using your measuring cups and spoons?” In the week that we cover mindfulness, the message states, “Breathe, Breathe deeply. You CAN savor each bite.” At the end of the six-month program, MFG participants receive six monthly newsletters to review and support concepts related to integrating and maintaining healthy lifestyle habits. They also receive supportive weekly text messages until the final data collection.

#### Moving Forward Self-Guided Program (SG)

Participants randomized to the SG receive a program binder that is divided into 26 sections, with each section addressing the topics listed in Table 1. Within each section is a brief guide to the topic and accompanying worksheets, handouts or activities to complete. Administrative study staff (not intervention staff) meet briefly (approximately 15 min) with the SG participants once individually to give them their binder and to provide an orientation to its layout and contents. Staff follows up with a monthly check-in phone call. At the end of the six months, SG participants also receive the monthly newsletters for six months until the final follow-up data collection.

### Randomization

Approximately one week before the intervention begins at each site, women who completed the baseline visit are randomized in a single block. The allocation assignments for each site are generated using a SAS program written by the data analyst, who has no contact with participants.

A few participants may be unable to complete the baseline visit before the main randomization round. In these cases, the data analyst prepares sealed, numbered envelopes containing the next allocation assignments for the site. As each woman completes her baseline visit, she is assigned the next envelope in the series.

### Statistical analyses

#### Power computations

Calculations were conducted for hierarchical longitudinal designs with differential attrition rates using software provided by Roy et al. [79] Results showed that an initial sample of 240 participants is sufficient to detect an effect size of 0.4, assuming attrition of about 10 % at each time point with power of 0.8 for a two -tailed test. Power will increase with the inter-correlation of observations over time, which we estimated conservatively at 0.4. The addition of covariates to the model will also increase power.

#### Data analysis

For a two-group randomized trial with repeated measures the mixed (multilevel) model is the preferred mode of data analysis [80]. With only two time points, the analysis reduces to a test of the difference between treatment groups in gains or losses in the outcome variable between baseline and 6-month follow up. The model easily generalizes to three time points (including the 12-month visit), in which case time can be indexed in simple linear fashion or represented either in quadratic form or via indicator variables for the first and second follow up measures. In that case, the analysis focuses on between group differences in outcome trajectories over time.

Our hypothesis is that subjects in the MFG condition will experience greater rates of weight loss, improved diet and physical activity patterns and other positive outcomes than subjects in the SG group. A major advantage of casting data analysis in the framework of the generalized linear (regression) model is that it easily accommodates covariates. One important covariate is time since diagnosis, which will be included in all analyses. A potentially important aspect of our modeling will be the investigation of dose–response effects. We will have data on frequency and pattern of attendance for each subject, which will allow us to model outcomes as a function of intensity of treatment.

## Discussion

This novel study examines the efficacy of a community-based weight loss program for African-American breast cancer survivors. Few behavioral interventions have targeted this high-risk population. Observational data highlight unhealthy eating and sedentary physical activity patterns among African-American breast cancer survivors, while qualitative data document their interest in making lifestyle changes to lose weight [62, 81]. Unfortunately, many survivors (regardless of race) are confused by the various dietary and physical activity recommendations, struggle with effective weight loss strategies, and relate a need for more structured programming [62]. Urban African-American women face further barriers in their quest to practice healthy lifestyles. Many African-Americans in the general population live in economically stressed neighborhoods where access to fresh fruits and vegetables may be limited or cost prohibitive, but cheaper high-fat foods are easily accessible [82, 83]. In addition, opportunities for physical activity in disadvantaged communities are often limited by a lack of safe open spaces, sidewalks in disrepair, gang violence, poor lighting, and insufficient police [84]. Focus groups with African-American breast cancer survivors highlight other important barriers, as well as facilitators. Barriers include pain, family commitments and lack of social support; facilitators include faith and spirituality, family and friend support, and desire to reduce overall health risks and risk of recurrence [62, 81]. If lifestyle change interventions are to be successful, barriers and facilitators must be addressed.

The Moving Forward intervention trial was developed in response to the recognized need for comprehensive weight loss programs that integrate cognitive-behavioral strategies related to lifestyle changes and the unique psychosocial needs of African-American breast cancer survivors. Recruitment and retention can be challenging for research targeting minority communities. To address this, the study team participates in a number of activities, such as health fairs, breast cancer survivor groups, health ministries, radio programs and educational forums within the African-American community. An important goal is to provide meaningful connections and information that meet the needs of this chronically underserved community. It is understood that regardless of whether women join the study, we are taking steps to build knowledge and trust to facilitate participation in health promoting activities and even future research studies that may benefit the women, their friends or families. Once women are involved in the study, we are particularly mindful of maintaining ongoing communication, as well as relating the value of their research participation. In addition, we prioritized conducting the program within the community to promote sustainability and to facilitate study retention by helping women from the same neighborhood connect with other breast cancer survivors.

Study data will contribute to a better understanding of the impact of weight loss on behavioral, psychosocial and biological outcomes among African-American breast cancer survivors. Unique to this study is its measurement of body composition and biological markers for both overall health and breast cancer recurrence risk. Previous weight loss interventions with African-American women, in general, report relatively small amounts of weight loss [85]. It is not clear if these losses correspond to equally small changes in body composition and biological markers of overall health and breast cancer recurrence. Results will improve our understanding of how behavior change and weight loss affect pathways associated with breast cancer recurrence and chronic disease risk, as well as those associated with quality of life and symptoms. If the intervention is successful, identifying optimal channels for dissemination will be critical.

## Abbreviations

CRP: C-reactive protein
IL-6: Interleukin-6
TNF-α: Tumor necrosis factor
IGF-1: Insulin-like growth factor-I
IGF-BP3: Insulin-like growth factor-binding protein-3
DXA: Dual energy x-ray
MFG: Moving Forward guided weight loss intervention
SG: Moving Forward self-guided weight loss intervention
BMI: Body Mass Index
FFQ: Food frequency questionnaire
PROMIS: Patient-reported outcomes measurement information system
SCT: Social Cognitive Theory
SEM: Socio-Ecological Model
